# The impact of 3D real-IR delayed post gadolinium MRI parameterisation on the diagnostic performance and optimal descriptor selection in Ménière’s disease

**DOI:** 10.1007/s00330-024-11218-0

**Published:** 2024-12-03

**Authors:** Steve Connor, Irumee Pai, Philip Touska, David Price, Sebastien Ourselin, Joseph V. Hajnal

**Affiliations:** 1 School of Biomedical Engineering and Imaging Sciences, King’s College London, London, UK; 2 Department of Neuroradiology, King’s College Hospital, London, UK; 3 Department of Radiology, Guy’s Hospital and St Thomas’ Hospital, London, UK; 4 Department of Ear, Nose and Throat Surgery, Guy’s and St Thomas’ Hospital, London, UK; 5 MRI Physics, Guy’s Hospital and St Thomas’ Hospital, London, UK

**Keywords:** Magnetic resonance imaging, Endolymphatic hydrops, Ear (inner), Perilymph endolymph

## Abstract

**Objectives:**

To compare the performance and optimal combination of MRI descriptors used for the diagnosis of Ménière’s disease (MD) between a real-IR sequence with “zero-point” endolymph (ZPE), and an optimised real-IR sequence with negative signal endolymph (NSE).

**Materials and methods:**

This retrospective single-centre cross-sectional study evaluated delayed post-gadolinium ZPE and NSE real-IR MRI in consecutive patients with Ménièriform symptoms (8/2020–10/2023). Two observers assessed 14 MRI descriptors. “Definite MD” (2015 criteria) and “all MD” ears (wider clinical criteria) were compared to controls. Cohen’s kappa and risk ratios (RR) were evaluated for each descriptor. Forward stepwise logistic regression established which combination of descriptors best predicted MD.

**Results:**

The study included 132 patients (57 men; mean age 57.7 ± 13.6), with 87 “all MD” (56 “definite”) and 39 control ears. The NSE sequence demonstrated increased perilymph SNR, and improved both diagnostic performance and reliability for 9/14 descriptors. However, ZPE demonstrated superior diagnostic performance for the best descriptor of “saccule absent, large as or confluent with the utricle” (RR 6.571, ZPE; 6.300, NSE) and that of “asymmetric perilymphatic enhancement” (RR 3.628, ZPE; 2.903, NSE). Both sequences combined these two descriptors in the optimal predictive model for “definite MD”, with “grade 2 cochlear hydrops” also significant for NSE. ZPE and NSE descriptor combinations both correctly classified 95.8% of ears. The ZPE descriptor combination performed better for “all MD” (ZPE, AUC-ROC 0.914; NSE, AUC-ROC 0.893).

**Conclusion:**

Parameter optimisation with NSE Real-IR influenced the optimal selection of MRI descriptors but did not improve their diagnostic performance in definite MD.

**Key Points:**

***Question*** *Delayed post-gadolinium ZPE (FLAIR) and NSE (REAL-IR) sequences are widely applied for diagnosing MD, but their relative benefits remain unclear*.

***Findings*** *Optimised NSE sequences improve perilymphatic depiction and influence the selection of the optimal MRI descriptors, but do not improve diagnostic performance*.

***Clinical relevance*** *Radiologists may continue to apply either ZPE or NSE sequences since they offer similar diagnostic abilities, but the choice of the sequence will influence which MRI features should be evaluated to support the diagnosis of MD*.

## Introduction

Ménière’s disease (MD) is an inner ear condition typified by the presence of progressive and fluctuating sensorineural hearing loss (SNHL), tinnitus and aural fullness. The structural correlate is considered to be an expansion of the endolymphatic space (ES) termed endolymphatic hydrops (EH) [1]. Magnetic resonance imaging (MRI) of EH in vivo is enabled by the permeability of the blood-perilymph barrier to gadolinium-based contrast agents (GBCAs), such that the enhancing perilymphatic space (PS) is separately depicted from the non-enhancing ES.

Since initial reports of EH visualisation with delayed post-intravenous GBCA MRI [2], there has been an evolution of methods used to optimise the signal from low-concentration GBCA within the perilymph. Altered parameterisation of three-dimensional fast spin echo (3D FSE) inversion recovery (IR) sequences has achieved increased perilymphatic signal through both the use of a long repetition time (TR) [3] and the application of modified 3D FSE sequences with very long echo trains and increased echo time (TE) [3–5]. A further development has been the ability to differentiate the endolymphatic signal from that of the surrounding temporal bone on MRI. The improved perilymphatic signal has now made it possible to use a single IR sequence with real reconstruction (real-IR) to distinguish negative signal endolymph (NSE) from intermediate signal bone and positive signal perilymph [6].

The diagnosis of MD with delayed post-gadolinium MRI is primarily based on the qualitative assessment of various MRI descriptors. The highest-performing MRI descriptors of MD have been shown to be saccular abnormalities [7, 8] and asymmetric perilymphatic enhancement (PLE) in recent cross-sectional studies [7–9] however these studies applied IR sequences with a time-to-inversion (TI) of endolymph designed to lie at the “zero-crossing point”, so depicting endolymphatic signal similar to bone. The impact of using MRI sequences which differentiate NSE on the diagnostic ability and optimal choice of MRI descriptors remains unknown. Understanding the relative benefits of “zero-crossing point” or NSE IR sequences would help radiologists select the optimal sequence parameters to apply and the best MRI features to interpret in order to diagnose MD.

The primary aim of this study was to compare the reliability and diagnostic performance of a range of MRI descriptors used for the diagnosis of MD between two different delayed post-gadolinium 3D-IR sequences with real reconstruction: a sequence with “zero-crossing point” endolymph (ZPE) and an optimised sequence with increased TR and NSE. Furthermore, we aimed to determine the optimal combination of MRI descriptors required to predict definite MD ears and a wider clinical spectrum of MD ears with each sequence.

## Methods

### Patients

**Fig. 1:**
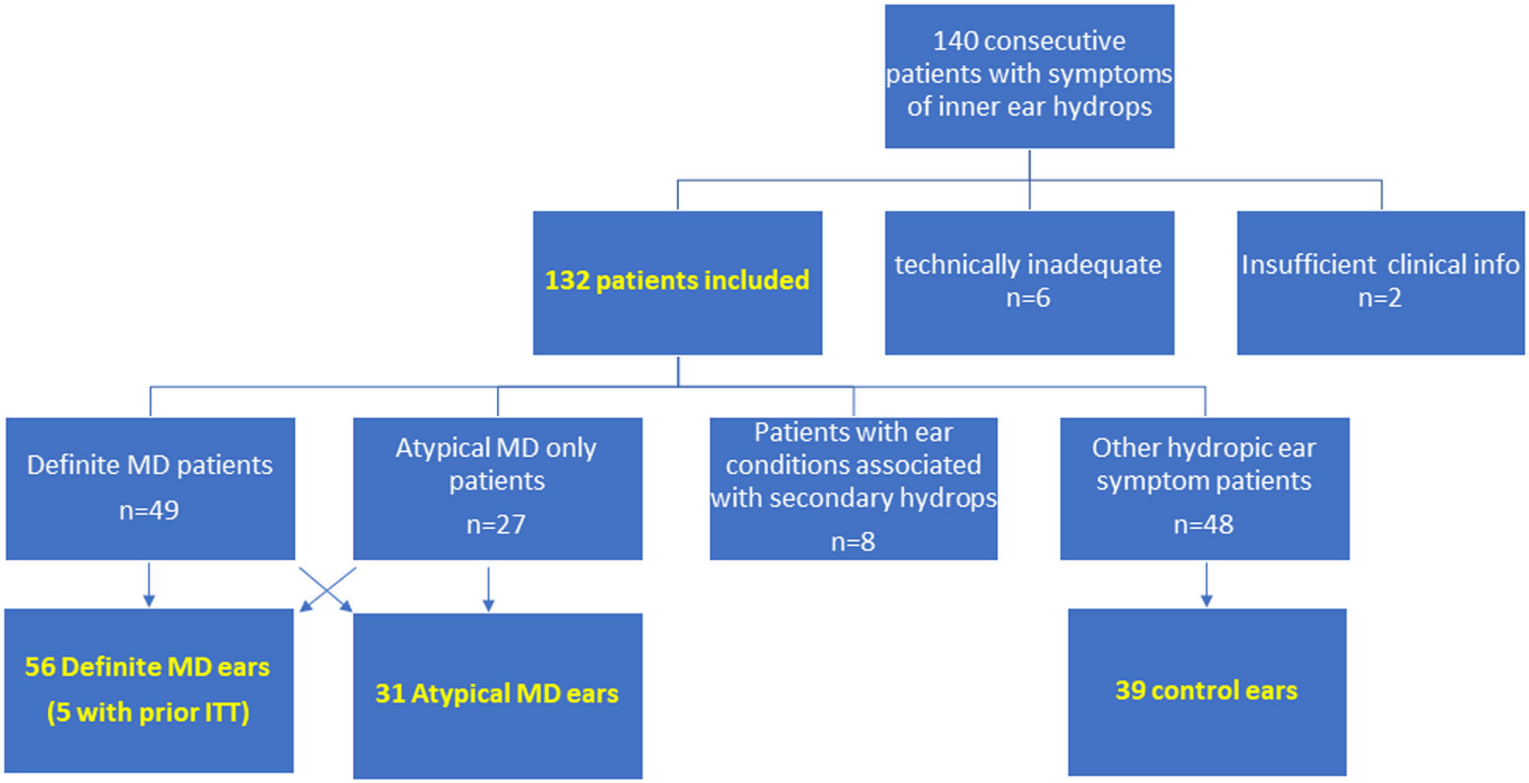
Flowchart demonstrating the selection process for 56 MD, 31 atypical MD and 39 control ears

The study was approved by the institutional ethical committee (GSTT Electronic Record Research Interface, IRAS ID: 257283, rec reference: 20/EM/0112). This was a retrospective cross-sectional study from a single institution. Consecutive patients undergoing delayed post-gadolinium MRI between August 2020 and October 2023 were retrieved by searching the PACS system (Sectra AB) (Table 1). This comprised patients presenting with symptoms of inner ear hydrops including episodic vertigo, sudden-onset or fluctuating SNHL, aural fullness and tinnitus. A priori exclusion criteria were inadequate contemporary clinical details for diagnostic classification, previous inner ear operations, and technically inadequate MR imaging (Fig. 1).

**Table 1:** Summary of the demographics and MD diagnosis of the study sample

Sex	75 Women/57 men
Age	Mean 47.7 ± 13.6 years Median 46 years (range 17–79 years)
Duration of symptoms	Mean 88.6 ± 91.9 months Median 47 months (range 1–360 months)
MD diagnosis	Unilateral definite MD 40 ears (22 right and18 left) Bilateral definite MD 7 ears Unilateral definite with contralateral atypical MD 2 ears (both left) Unilateral atypical MD 25 (11R and14L) (see Supplementary Table 4 for subtypes) Bilateral atypical MD 2 Total 56 definite MD ears Total 31 atypical MD ears

### Clinical data and classification

Two observers (S.C., I.P.) reviewed the contemporary clinical and audiometric data by consensus, blinded to imaging findings. Clinical review was performed in the six months and audiometry in the twelve months prior to the MRI study. “Definite MD” was classified according to the current 2015 Barany Society criteria [10]. A wider clinical spectrum of “all MD” also included ears defined by additional clinical classifications (“atypical MD”) [10–15]. MD was not diagnosed if there was a clinical diagnosis of secondary hydrops. Control ears were obtained from patients without MD vertigo or any “all MD” criteria in either ear, and with either normal hearing or isolated high-frequency SNHL (Supplementary Table 1). Intra-tympanic therapy (ITT) with gentamicin or corticosteroid prior to the MRI study was recorded.

### MRI protocol and technique

MRI for inner ear EH was performed on a 3-Tesla Siemens Magnetom® Skyra scanner with a 64-channel head coil. MRI was performed 3.5–4.5 h after the intravenous administration of gadoterate meglumine (0.2 mmol/kg). Two high-resolution 3D IR sequences with real reconstruction (3D real-IR) were acquired consecutively. The first sequence (ZPE) with TR, 6000 ms; TE, 180 ms; TI, 2000 ms resulted in endolymph being at the zero-crossing point at time TI, with the zero-crossing point endolymph having similar pixel values as bone. The second sequence (NSE) was optimised for increased perilymphatic signal, with TR, 15,130 ms; TE, 550 ms; and TI, 2700 ms and since endolymph was below the zero-crossing point at time TI, it demonstrated NSE which was a lower signal than bone (Supplementary Fig. 1). An additional T2-weighted sampling perfection with application-optimised contrasts using different flip angle evolution (SPACE) sequences was performed. Siemens product sequences were used with parameters tabulated in Table 2.

**Table 2:** MRI parameters for the zero-crossing point endolymph (ZPE) and optimised negative signal endolymph (NSE) 3D-real IR sequences and the T2-SPACE sequence

	Zero point endolymph (ZPE) 3D real-IR	Optimised negative signal endolymph (NSE) 3D real-IR	T2-SPACE
TR	6000 ms	15,130 ms	1000 ms
TE	180 ms	550 ms	125 ms
Inversion time	2000 ms	2700 ms	NA
Number of excitations	1	1	2
Refocusing flip angle	180° (constant)	130° (constant)	100°
Pixel bandwidth	220	435	255
Echo train length	27	267	52
Pixel spacing	0.7 mm	0.66 mm	0.31 mm
Slice thickness	0.7 mm	0.6 mm	0.3 mm
Matrix size	256 × 240	320 × 270	262 × 512
Field of view	190 × 178 mm	210 × 177 mm	80 × 160 mm
Acquisition time	13.38 min	11.21 min	6.38 min

### Extraction and definition of qualitative MRI descriptors

**Fig. 2:**
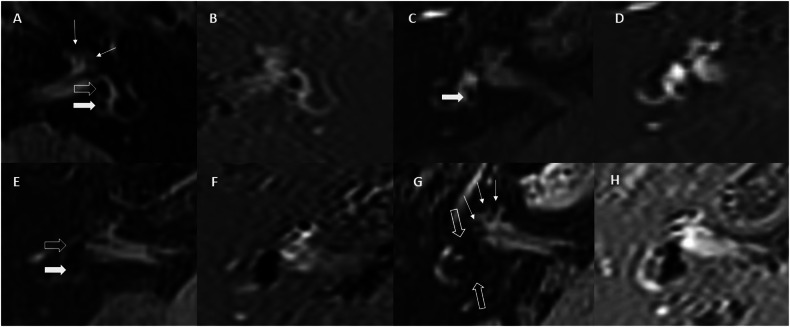
Zero point endolymph (ZPE) 3D real-IR (**A**, **C**, **E**, and **G**) and corresponding optimised NSE real-IR (**B**, **D**, **F**, and **H**) delayed post gadolinium images demonstrate the key saccule and other MRI descriptors in patients with MD. Images illustrate saccule to be larger than the utricle (**A**, **B**), saccule absent (**C**, **D**), as well as saccule and utricle confluence (**E**, **F**). The anteriorly located saccule is indicated by the open arrow and the posteriorly located utricle by a filled arrow (**A**–**F**). Note how the nodular low signal of the bony intercalar septum on ZPE (arrows in a) may mimic cochlear grade 1 hydrops, whereas the differential signal of bone and endolymph with NSE results in this descriptor being more reliable. Cochlear grade 2 hydrops are clearly depicted on both sequences and have high reliability (arrows in **G**, **H**). When there is severe vestibular EH and the PS is not visible, then endolymph cannot be delineated from the surrounding bone on ZPE (open arrow in **G**) whereas it can be demonstrated separately with NSE (**H**)

MRI grading scales or unique individual EH descriptors which had been previously applied to ≥ 3 published diagnostic accuracy studies of MD were collated. This yielded four grading scales (Nakashima, Barath, Bernaerts, and Kahn) (Supplementary Table 2) describing 12 MRI descriptors, and the two additional individual descriptors of asymmetric peri-lymphatic enhancement (PLE) and an absent saccule [7, 8, 16–21]. The 14 MRI descriptors are listed in Table 3 and described in Supplementary Table 3 and Fig. 2. Only one definition of cochlear grades was applied [7] since they differ little between scales. Confluence with the utricle may preclude evaluation of other saccule descriptors, so saccule descriptors were combined to avoid missing values.

**Table 3:** Inter-rater reliability Kappa values, sensitivity/specificity, risk-ratios for the 14 MRI descriptors with ZPE and NSE sequences

	Cohens Kappa (95% CI) ZPE	Cohens Kappa (95% CI) NSE	Sensitivity/specificity risk ratio (95% CI) for definite MD diagnosis ZPE	Sensitivity/specificity/risk ratio (95% CI) for definite MD diagnosis NSE
**Cochlear**				
Grade ≥ 1 cochlear hydrops	***κ*** = **0.666 (0.574–0.758)** ***p*** < **0.001**	***κ*** = **0.788 (0.740–0.864)**	**87.5/74.3 4.753 (2.642–4.753)**	**91.1/71.8 4.782 (2.745–8.331)**
Grade 2 cochlear hydrops	*κ* = 0.967 (0.930–0.100)	*κ* = 0.957 (0.916–0.998)	**80.4/97.4 4.358 (2.585–7.346)**	**83.9/100 5.333 (2.960–9.611)**
Asymmetric PLE*	*κ* = 0.834 (0.758–0.911)	*κ* = 0.917 (0.860–0.974)	Standardised windowing 75.0/97.4 3.628 (2.313–5.691) Equivalent windowing 75.0/92.3 3.333 (2.123–5.234)	Standardised windowing 67.9/94.9 2.903 (1.974–4.268) Equivalent windowing 69.6/89.7 2.774 (1.857–4.145)
**Superior vestibule**				
> 33% VES area relative to TV area (superior)	***κ*** = **0.693 (0.597–0.789)**	***κ*** = **0.786 (0.712–0.860)**	80.4/79.5 **4.890 (2.519–9.494)**	83.9/74.4 **4.350 (2.175–8.387)**
> 50% VES area relative to TV area (superior)	*κ* = 0.881 (0.805–0.957)	*κ* = 0.934 (0.877–0.991)	66.1/100 2.950 (2.066–4.213)	69.6/97.4 3.154 (2.118–4.698)
No lateral SCC ampullary PS visible	***κ*** = **0.788 (0.623–0.953)**	*κ* = 0.905 (0.776–1.00)	26.8/100 1.951 (1.576–2.416)	17.9/100 1.848 (1.519–2.247)
Lateral SCC posterior limb ES extension	*κ* = 0.802 (0.688–0.916)	*κ* = 0.883 (0.799–0.967)	42.9/100 2.219 (1.716–2.868)	51.8/100 2.444 (1.829–3.267)
PS not visible (superior)	*κ* = 0.849 (0.708–0.990)	***κ*** = **0.772 (0.629–0.915)**	21.4/100 *1.886 (1.541–2.310)*	35.7/100 *2.083 (1.646–2.637)*
**Inferior vestibule**				
Saccule large as or confluent with the utricle	*κ* = 0.951 (0.908–0.994)	*κ* = 0.943 (0.898–0.988)	**76.8/100 4.000 (2.498–6.405)**	*75.0/97.4 3.628 (2.313–5.691)*
Saccule absent, large as or confluent with the utricle	*κ* = 0.933 (0.888–0.978)	*κ* = 0.943 (0.902–0.984)	**87.5/100 6.571 (3.322–12.999)**	**87.5/97.4 6.300 (3.186–12.459)**
Saccule confluent with the utricle	*κ* = 0.940 (0.887–0.993)	*κ* = 0.978 (0.947–1.00)	*73.2/100 3.600 (2.342–5.535)*	**78.6/100 4.250 (2.591–6.971)**
> 50% VES area relative to TV area (inferior)	*κ* = 0.963 (0.923–1.00)	*κ* = 0.966 (0.927–1.00)	69.6/100 3.294 (2.215–4.898)	**78.6/100 4.250 (2.591–6.971)**
PS not visible (inferior)	*κ* = 0.857 (0.745–0.969)	***κ*** = **0.762 (0.627–0.897)**	28.6/100 1.975 (1.589–2.455)	42.9/100 2.219 (1.716–2.868)
VES contacting the oval window	*κ* = 0.890 (0.820–0.960)	*κ* = 0.83 (0.746–0.914)	69.6/100 3.294 (2.215–4.898)	73.2/97.4 3.449 (2.241–5.308)

Kappa values < 0.8 and risk ratio > 4 in bold

*PLE* perilymphatic enhancement, *VES* vestibular endolymphatic space, *SCC* semi-circular canal, *PS* perilymphatic space, *TV* total vestibular

^*^There were 12 patients with asymmetric cochlear PLE only recorded on the ZPE sequence when using standardised windowing and 8 patients when using equivalent windowing, whereas there were 2 patients with asymmetric cochlear PLE only recorded on the NSE sequence when applying both standardised and equivalent windowing

### Imaging analysis

**Fig. 3:**
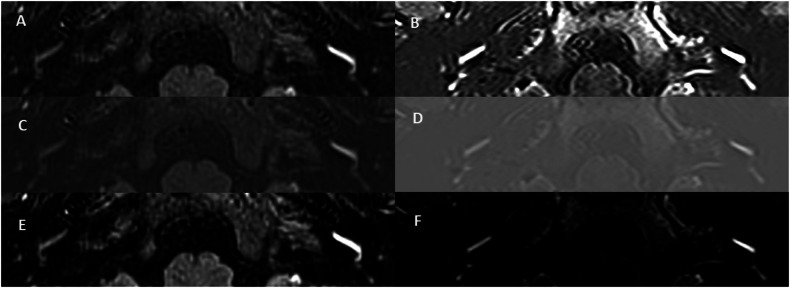
Delayed post gadolinium images through the cochlear basal turns of unilateral definite MD patients indicating the evaluation of asymmetric PLE with the different windowing algorithms and methods. Zero point endolymph (ZPE) 3D real-IR (**A**, **C**, and **E**) and corresponding optimised NSE real-IR (**B**, **D**, and **F**) images with histogram matching algorithm (**A**, **B**), min–max algorithm (**C**, **D**) and equivalent window settings (**E**, **F**) in a patient with left-sided MD. Clear asymmetric PLE was noted with ZPE on both standardised window settings, whereas there is no asymmetric PLE demonstrated with NSE when applying the histogram algorithm (**B**) and only equivocal asymmetry when applying the min–max algorithm (**D**). The equivalent windowing method was applied with both sequences (**E**, **F**) which was centred at the normal cochlear perilymph signal (172 for ZPE and 290 for NSE) and with the width of the ZPE histogram algorithm (382). This more clearly demonstrated left asymmetric PLE with NSE (**F**). The signal ratio between the MD and the normal contralateral ear was 3.0 with ZPE and 1.6 with NSE

Two radiologists (P.T. and S.C.), with 7 years and 25 years of subspecialty head and neck radiology experience, independently evaluated ZPE and NSE sequences in isolation and at least two months apart in a random order, whilst blinded to clinical information. Images were viewed with fixed magnification on a PACS workstation (Sectra workstation, Sectra AB), with T2w SPACE images used for anatomical correlation. The qualitative MRI descriptors were analysed according to the defined criteria (Supplementary Table 3) and the four grading scales were also scored. The images were reviewed with optional min–max intensity normalisation or histogram matching PACS-based standardised algorithms, according to which better depicted the distribution of the ES. The two observers achieved consensus when different scores were obtained. A post hoc analysis of asymmetric cochlear PLE was performed with an equivalent window width for both sequences, with the window level at the signal intensity of normal PLE and the width matched to the histogram matching algorithm on ZPE (Fig. 3).

**Fig. 4:**
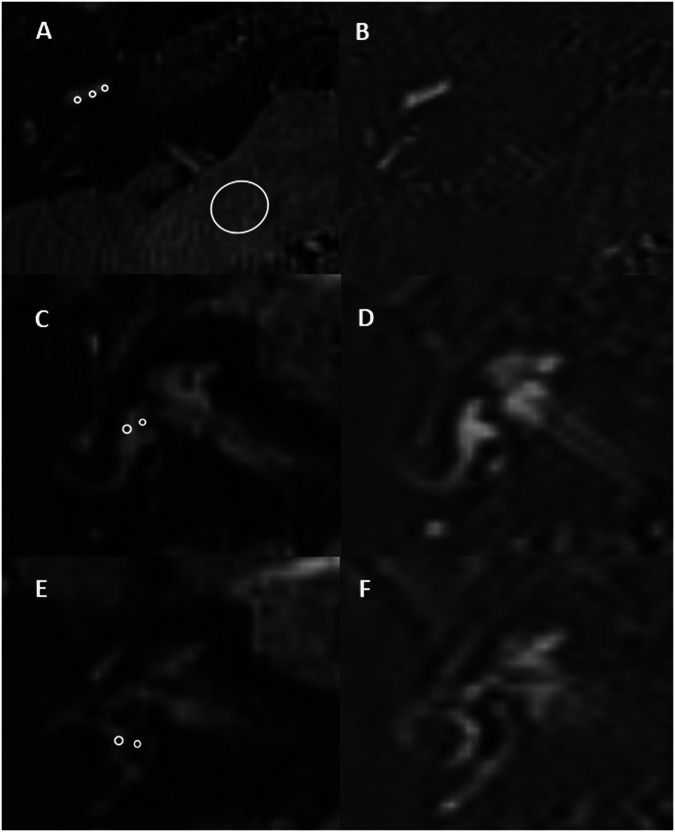
Zero point endolymph (ZPE) 3D real-IR (**A**) and corresponding optimised NSE real-IR (**B**) delayed post gadolinium images through the inferior segment of the cochlear basal turn. Cochlear perilymphatic signal intensity was measured (figure) as the mean of three 1 mm^2^ regions of interest (ROIs) placed within the scala tympani of the basal turn (small circles). A 30 mm^2^ ROI was placed in both middle cerebellar peduncles (large circle) as an internal reference for the signal intensity ratio. ZPE and corresponding NSE images through the inferior vestibule (**C**, **D**) and the superior vestibule (**E**, **F**) in an ear without EH. The vestibular perilymphatic signal was quantified from the mean of two 0.7 mm^2^ ROIs, one placed lateral to the location of the saccule (larger circle in **C**) and one lateral to the utricle (larger circle in **E**), or alternatively within the adjacent ampullae in the presence of severe EH. The vestibular endolymphatic signal was evaluated from the mean of two 0.5 mm^2^ ROIs, one placed in the saccule (smaller circle in **C**) unless absent, and one in the utricle (smaller circle in **E**). A mean of the values obtained by the two observers was used for further analysis

Cochlear and vestibular perilymphatic signal intensity was measured as the mean of multiple standardised regions of interest (ROI) (Fig. 4). An internal reference was obtained from the mean of 30 mm^2^ ROIs placed in both middle cerebellar peduncles [9] with perilymphatic signal intensity ratio (SIR) calculated as perilymph signal/middle cerebellar peduncle signal. A 5 mm^2^ ROI was placed within artefact-free air adjacent to each pinna, with a perilymphatic signal-to-noise ratio (SNR) defined as perilymph signal/ mean σ air signal and perilymphatic to endolymphatic contrast to noise ratio (CNR) as perilymphatic-endolymphatic signal/ mean σ air signal.

### Statistical analysis

Statistical analyses were performed using SPSS® Statistics 27.0 (IBM®).

Inter-rater reliability of individual descriptors and grading scales were evaluated with Cohen’s kappa test and a weighted kappa (κw*),* respectively. The inter-rater reliability of cochlear SIR was evaluated with an intra-class correlation coefficient (ICC).

The association of MRI descriptors between sequences was evaluated with the chi-squared test, and Phi and Cramer’s *V* coefficients were calculated to indicate the strength of the association. Cochlear SIR was not normally distributed (Shapiro–Wilk’s test; *p* < 0.05) so was compared using Wilcoxon signed-rank test. The ratio of cochlear PLE between unilateral “definite MD” ears and the contralateral ears was also compared between sequences.

Sensitivity, specificity and the risk ratio (probability) of a “definite MD” ear in the presence of each MRI descriptor were calculated. Chi-squared test of independence compared the scores for the four grading scales and likelihood ratios were calculated.

A binomial forward stepwise logistic regression was performed to determine the optimal combination of MRI predictors of “definite MD” and “all MD” for ZPE and NSE. Descriptors with reliability kappa score < 0.8 and variance inflation factor > 5 were excluded. According to Vittinghoff and McCulloch [22], the sample size allowed the inclusion of all the MRI-based predictors in the model. The area under the receiver-operating characteristic (AUC-ROC) curve was calculated. The forward stepwise logistic regression was repeated after removing MD ears which had undergone ITT and with the use of post hoc equivalent windowing for PLE.

## Results

### Descriptive data for cohort

Of the 140 consecutive patients referred for delayed post-gadolinium MRI. Patients with technically inadequate MR imaging (*n* = 6) and insufficient clinical information (*n* = 2) were excluded (Fig. 1). The remaining 132 patients (75 female, 57 males; age mean 57.7 ± 13.6 years; duration of symptoms mean 88.6 ± 91.9 months) comprised 49 patients with definite MD (7 bilateral), 27 patients with atypical MD alone and 8 ears with secondary hydrops (Table 1 and Supplementary Table 4). There were 56 definite MD ears, 31 atypical MD ears, and 39 control ears.

### Inter-rater reliability

The inter-rater reliability for the MRI descriptors and grading scales (*n* = 264) is documented in Table 3 and Supplementary Table 5. The NSE sequence achieved higher kappa values for 9/14 descriptors. The lowest kappa values were recorded for “grade ≥ 1 cochlear hydrops” (κ = 0.666) and “> 33% VES relative to the total vestibular (TV) area (superior)” (κ = 0.693) with the ZPE sequence. Both sequences achieved κ > 0.9 for all saccule-based descriptors and grade 2 cochlea.

The weighted kappa (κw) values indicated excellent reliability for all the grading scales when evaluated on both sequences (Supplementary Table 5). There was greater reliability for the evaluation of cochlear perilymph SIR with the ZPE compared to the NSE sequence with an ICC of 0.971 (95% CI: 0.929–0.955) vs 0.819 (95% CI: 0.752–0.858).

### Comparison of MRI descriptors and quantitative measures between the ZPE and NSE

There was a significant association between the presence of all MRI descriptors and grading scales (Supplementary Table 5) for the two sequences (*n* = 264; *p* < 0.001) (Supplementary Table 6), and there was a particularly strong association for “grade 2 cochlear hydrops” (φ = 0.915).

The median perilymph: endolymph CNR, cochlear perilymph SNR and vestibular perilymph SNR were increased with the NSE sequence (175.4, IQR 111.1; 186.3, IQR 106.3, and 303.9, IQR 141.9) compared to the ZPE sequence (90.0, IQR 51.2; 118.3, IQR 69.7, and 138.3, IQR 80.8). There was a statistically significant increase in median cochlear SIR with the NSE sequence (16.4; IQR 11.8) compared to the ZPE sequence (0.8; IQR 0.5) (*z* = 14.085, *p* < 0.001).

**Fig. 5:**
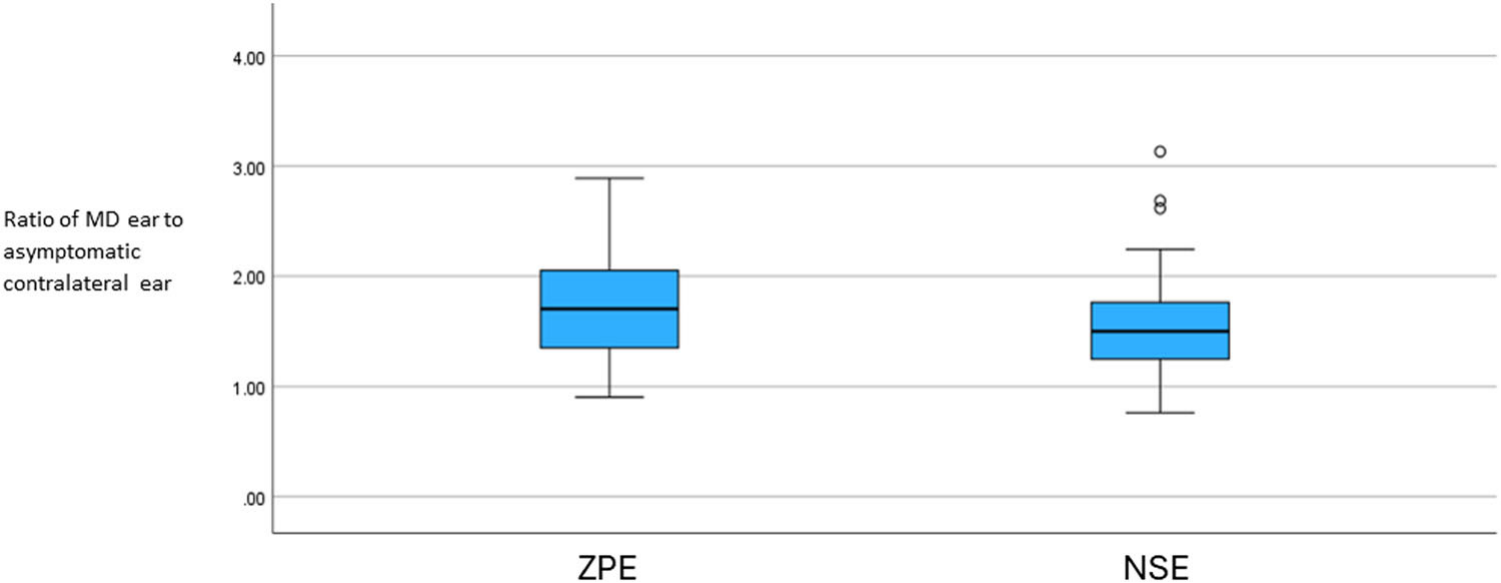
Ratio of cochlear PLE of the symptomatic MD ear to the asymptomatic ear in 40 patients with unilateral MD with zero-point endolymph (ZPE) 3D real-IR and optimised negative signal endolymph (NSE) 3D real-IR sequences

The cochlear PLE enhancement ratio between the affected MD ear and contralateral asymptomatic ear was increased with the ZPE sequence (1.7049; IQR 0.72) relative to the NSE sequence (1.5010; IQR 0.52) in the 40 patients with unilateral definite MD, although this was not statistically significant (*z* = 2.540, *p* = 0.11) (Fig. 5).

### Diagnostic performance of individual descriptors and parameters

The sensitivity, specificity and risk ratio (probability) of a definite MD ear in the presence of each MRI descriptor is demonstrated in Table 3 and the likelihood ratios with each grading scale are shown in Supplementary Table 5.

The NSE demonstrated increased sensitivity for 10/14 MRI descriptors and risk ratio for 9/14 descriptors. The risk ratio for asymmetric PLE was increased with both optional and equivalent windows (3.628, 3.333 ZPE vs 2.908, 2.774 NSE).

The highest risk ratio was achieved by “saccule absent, as large as or confluent with the utricle” for both sequences (6.571, ZPE; 6.300, NSE).

### Logistic regression

**Fig. 6:**
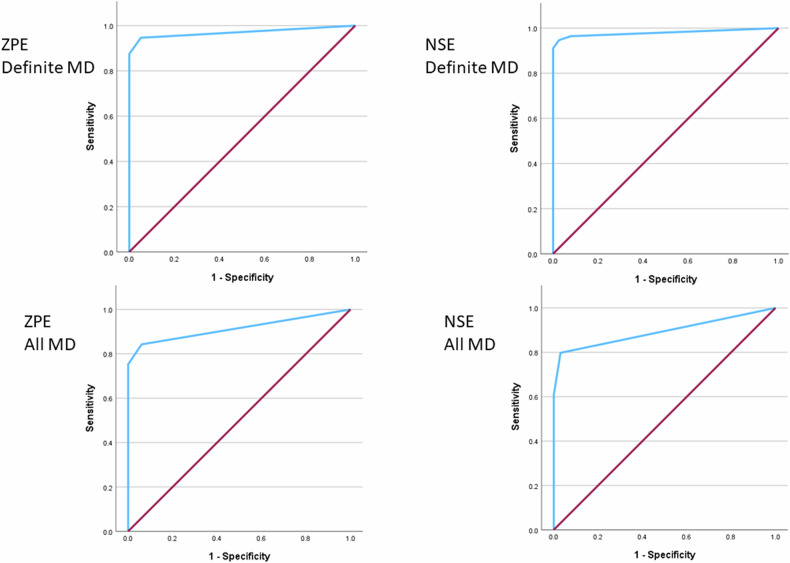
ROC curves for the optimal models to predict definite MD and all MD ears with zero-point endolymph (ZPE) 3D real-IR and optimised negative signal endolymph (NSE) 3D real-IR sequences

The performance of optimal MRI descriptor combinations for the prediction of definite MD with ZPE and NSE sequences are demonstrated in Table 4 and Fig. 6.

**Table 4:** Number of ears and MRI descriptors entered into logistic regression models, with the significant MRI descriptor combinations and their diagnostic performance using ZPE and NSE sequences

	Number of MD/ control ears	Number of MRI descriptors included in analysis^†^	Statistically significant MRI descriptors	Significance of MRI descriptor in model^*^	*R*^2^^#^	% Cases correctly classified	Sens/spec	PPV/NPV	AUC-ROC (95% CI)
**ZPE**									
Definite MD	56/39	8	1) Saccule absent, large as or confluent with the utricle 2) Asymmetric cochlear PLE	χ^2^ = 89.405 *p* < 0.001 χ^2^ = 12.766 *p* = 0.001	88.8%	95.8%	94.6%/ 7.4%	98.1%/ 92.7%	0.972 (0.937–1.000)
Definite MD without ITT	51/39	8	1) Saccule absent, large as or confluent with the utricle 2) Asymmetric cochlear PLE	χ^2^ = 87.821 *p* < 0.001 χ^2^ = 14.455 *p* = 0.001	91.1%	96.7%	96.1%/97.4%	98.0%/ 95.0%	0.979 (0.947–1.000)
Definite MD with equivalent windowing for PLE	56/39	8	1) Saccule absent, large as or confluent with the utricle 2) VESCO 3) Asymmetric cochlear PLE	χ^2^ = 89.405 *p* < 0.001 χ^2^ = 8.078 *p* < 0.001 χ^2^ = 3.274 *p* = 0.001	88.1%	94.7%	91.1%/100%	100%/ 95.1%	0.970 (0.933–1.000)
All MD	83/39	8	1) Saccule absent, large as or confluent with the utricle 2) Asymmetric cochlear PLE	χ^2^ = 68.403 *p* < 0.001 χ^2^ = 11.218 *p* < 0.001	69.6%	87.7%	84.3%/97.0%	98.7%/ 69.7%	0.914 (0.864–0.963)
**NSE**									
Definite MD	56/39	7	1) Saccule absent, large as or confluent with the utricle 2) Grade 2 cochlear hydrops 3) Asymmetric cochlear PLE	χ^2^ = 79.935 *p* < 0.001 χ^2^ = 21.498 *p* < 0.001 χ^2^ = 3.562 *p* = 0.059	90.2%	95.8%	94.6%/97.4%	98.1%/ 92.7%	0.979 (0.950–1.000)
Definite MD without ITT	51/39	7	1) Saccule absent, large as or confluent with the utricle 2) Grade 2 cochlear hydrops 3) Asymmetric cochlear PLE	χ^2^ = 78.475 *p* < 0.001 χ^2^ = 23.064 *p* < 0.001 χ^2^ = 4.604 *p* = 0.059	92.9%	96.7%	96.1%/97.4%	98.0%/ 95.0%	0.979 (0.950–1.000)
Definite MD with equivalent windowing for PLE	56/39	7	1) Saccule absent, large as or confluent with the utricle 2) Grade 2 cochlear hydrops	χ^2^ = 79.935 *p* < 0.001 χ^2^ = 19.121 *p* = 0.001	88.5%	95.8%	95.6%/97.4%	98.1%/ 92.7%	0.971 (0.936–1.00)
All MD	83/39	7	1) Saccule absent, large as or confluent with the utricle 2) Grade 2 cochlear hydrops	χ^2^ = 59.013 *p* < 0.001 χ^2^ = 10.356 *p* = 0.001	63.0%	84.4%	79.8%/97.0%	98.6%/ 64.0%	0.893 (0.837–0.948)

*MD* Ménière’s disease, *NPV* negative predictive value, *PPV* positive predictive value, *AUC-ROC* area under the receiver operating characteristic curve, *ITT* intra-tympanic treatment, *PLE* perilymphatic enhancement

^*^At each step, the most highly correlated remaining variable was added, with a significance level set at *p* < 0.05 for a predictor to be entered into the model and *p* > 0.1 for it to be removed

^†^The MRI descriptors with kappa values < 0.8 and those with collinearity demonstrated by a variance inflation factor > 5 (“> 50% VES area relative to TV area (inferior)”, “saccule large as or confluent with the utricle”, “saccule confluent with the utricle”) were excluded from all the analyses. There were no standardised residuals with values of greater than 2.5 standard deviations (SDs)

^#^*R*^2^ summarises the proportion of variance in the diagnosis of MD associated with the MRI descriptor predictors with larger values indicating more variance. Nagelkerke’s *R*^2^ was calculated which adjusts the statistic to cover 0–1

For the prediction of definite MD with ZPE only “saccule absent, large as or confluent with the utricle” and “asymmetric PLE” were statistically significant, whilst “grade 2 cochlear hydrops” was also significant for NSE. The descriptor combinations for both sequences correctly classified 95.8% of ears with Nagelkerke *R*^2^ of 88.8% for ZPE and 90.2% for NSE. Repeat analysis with the exclusion of ITT cases or with the use of equivalent windowing methods for PLE had little impact on the AUC-ROC of each model (all AUC-ROC ≥ 0.97), although the latter influenced the significant MRI descriptors. When applied to “all MD” ears, “saccule absent, large as or confluent with the utricle” and “asymmetric PLE” were again significant descriptors with ZPE, whilst both the combined saccule-based descriptors and “grade 2 cochlear hydrops” were the significant NSE descriptors. The optimal model for “all MD” performed better with ZPE than NSE (AUC 0.914, Nagelkerke *R*^2^ 69.6% vs AUC 0.893, Nagelkerke *R*^2^ 63%) and “asymmetric PLE” was no longer a significant predictor for the wider clinical spectrum of “all MD” ears.

### Discussion

The optimised NSE real-IR sequence demonstrated superior reliability to the zero-point endolymph (ZPE) sequence for 9/14 MRI descriptors. The median perilymph: endolymph CNR and cochlear perilymph SNR were 1.9× and 1.6× higher with the NSE sequence. The NSE sequence also demonstrated higher risk ratios for “definite MD” diagnosis with 9/14 MRI descriptors. It was noted that the optimal descriptor of “saccule absent, large as or confluent with the utricle” demonstrated similar diagnostic performance (ZPE risk-ratio 6.571; NSE risk-ratio 6.300) and high specificity (100% and 97.4%) for the two sequences. Moreover, asymmetric cochlear PLE was more frequently recorded with ZPE, with higher risk ratios for MD diagnosis (3.628 vs 2.903) and with increased quantitative asymmetry in cochlear PLE demonstrated in unilateral MD. A forward stepwise logistic regression demonstrated “saccule absent, large as or confluent with the utricle” and “asymmetric PLE” to be the most statistically significant predictors of both “definite MD” and the wider clinical spectrum of “all MD” using the ZPE sequence. The optimal descriptor combinations differed for NSE, with “Grade 2 cochlear hydrops” being added to the saccular descriptor and “asymmetric PLE” in order to optimally diagnose “definite MD ears”, whilst “asymmetric PLE” was no longer a significant predictor for “all MD” ears. The optimal ZPE and NSE descriptor combinations both correctly classified 95.8% of the “definite MD ears”, whilst the optimal ZPE model performed better than NSE for “all MD ears” (AUC-ROC 0.914 vs AUC-ROC 0.893). Therefore, whilst modifications to the parameterisation of post gadolinium real-IR sequences increase the perilymphatic signal and enable the depiction of NSE hydrops in MD, this has little impact on its diagnostic ability, and radiologists should be aware that it will influence the key MRI features to analyse.

Previous studies have often referred to the NSE sequence under the umbrella term of REAL-IR whilst the ZPE sequence reproduces the signal characteristics of the frequently applied FLAIR sequence. Whilst a recent meta-analysis highlighted a potential improvement in the diagnostic performance of some MRI descriptors when using MRI techniques with NSE, the studies were heterogeneous and hence the pooled comparison was subject to bias [16]. Two previous studies by Zhao et al and Suarez Vega et al have compared similar optimised NSE 3D real-IR sequences to magnitude reconstructed ZPE (FLAIR) sequences, and these both demonstrated increased sensitivity with the NSE real-IR sequence [23, 24]. Although our data also demonstrated some improvement in sensitivity with NSE across a range of MRI descriptors, this was not the case for the two most significant combined saccular and “asymmetric PLE” descriptors. Apart from applying equivalent real-reconstruction methods across both sequences, our report differs from previous studies in other important respects. Firstly, our study design included a control group allowing optimal individual and combined MRI descriptor performance to be evaluated. Secondly, asymmetric PLE was included in the analysis as well as a wide range of MRI descriptors. Thirdly, a cohort with atypical MD was incorporated since the characterisation of hydropic ear disease in this wider clinical spectrum is increasingly recognised to be important [25].

“Asymmetric PLE” was an inferior qualitative and quantitative marker of MD with NSE. This is intriguing since the quantitative cochlear perilymphatic signal was increased with the longer TR NSE sequence and the T1w effects of this real-IR sequence would be expected to result in greater differential signal between cochleae with differing gadolinium concentrations. An alternative hypothesis is that there may be an impact from the relatively increased TE of the NSE sequence and hence its T2 weighting. If the perilymphatic fluid of an asymmetrically enhancing MD ear also demonstrates pathological T2-shortening, then this could result in a differential reduction in its signal ratio to the contralateral normal ear on the NSE sequence. Of note, the only previous study to compare cochlear PLE between long and short TE sequences did not demonstrate a reduction in asymmetric PLE with the long TE sequence [5] however this was in a smaller cohort of 22 patients.

There are limitations to this study which should be considered. Firstly, there are two aspects of the methodology which may result in systematic bias. The ZPE sequence was obtained immediately prior to the NSE sequence in all cases although both were performed within the 3.5–4-h window considered to be optimal for PLE [26]. In addition, there was a differential application of the two standardised windowing algorithms for optimal visualisation of the endolymphatic structures with the two sequences. In the case of asymmetric PLE, this was addressed by the additional application of equivalent window widths for both ZPE and NSE which resulted in similar outcomes to the standardised windowing. Secondly, there was innate bias in patient selection introduced by the case-controlled study design [27]. Thirdly, there were multiple differences in parameterisation between the ZPE and NSE sequences, including the longer TR, longer TE, longer echo train, smaller flip angle and TI designed for the visualisation of NSE, therefore making it difficult to dissect the most influential modifications. However, the key finding of no overall difference in diagnostic performance despite all these modifications remains particularly pertinent. Fourthly, there was an interval between the imaging index test and the clinical MD reference standard due to the retrospective study design. Fifthly, a double-dose gadolinium technique with gadoterate was employed in this study, and it is appreciated that single-dose studies are generally advocated, limiting the generalisation of our results. However, it should be noted that gadoterate is of lower T1 relaxivity than gadobutrol which is an agent advised for single-dose studies, so this partly mitigates any potential difference in perilymphatic SNR [28, 29]. Finally, although the MRIs were interpreted blinded to the clinical diagnosis, there was potential bias due to the unavoidable viewing of multiple MRI descriptors simultaneously within each ear.

In conclusion, optimisation of a delayed post-gadolinium NSE real-IR sequence improves the CNR and SNR as well as the reliability and diagnostic performance of most individual MRI descriptors. However, the most statistically significant parameters of “saccule absent, large as or confluent with the utricle” and “asymmetric PLE” performed better with ZPE. The optimal combination of MRI descriptors to diagnose MD differed between the two sequences. The best combinations of MRI descriptors for ZPE and NSE demonstrated a similar ability to predict definite MD ears, however, the optimal ZPE predictors were better able to predict a wider spectrum of MD ears. The relative advantages of each approach justify the continued application of either sequence in clinical practice, however, radiologists should appreciate that the choice of sequence will influence which MRI features should be evaluated for the diagnosis of MD.

## Supplementary information

ELECTRONIC SUPPLEMENTARY MATERIAL

## Abbreviations

AUC: Area under the curve
CI: Confidence interval
CNR: Contrast to noise ratio
EH: Endolymphatic hydrops
ES: Endolymphatic space
FSE: Fast spin echo
GBCA: Gadolinium-based contrast agent
IR: Inversion recovery
ITT: Intratympanic therapy
MD: Ménière’s disease
MRI: Magnetic resonance imaging
NSE: Negative signal endolymph
PLE: Perilymphatic enhancement
PS: Perilymphatic space
Real-IR: IR sequence with real reconstruction
ROC: Receiver operating characteristic
SNHL: Sensorineural hearing loss
SNR: Signal to noise ratio
SPACE: Sampling perfection with application of optimised contrasts using different flip angle evolution
TE: Echo time
TR: Repetition time
TV: Total vestibular
VES: Vestibular endolymphatic space
ZPE: Zero-point endolymph
