# Supplementary material for: The impact of 3D real-IR delayed post gadolinium MRI parameterisation on the diagnostic performance and optimal descriptor selection in Ménière’s disease
Source: Eur Radiol. 2024 Dec 3;35(3):1290–302. doi: 10.1007/s00330-024-11218-0 (PMC11836106; doi:10.1007/s00330-024-11218-0)
Supplement: Supplementary file 1 — ELECTRONIC SUPPLEMENTARY MATERIAL [file 330_2024_11218_MOESM1_ESM.docx]

| Clinical classification | Clinical criteria |
| --- | --- |
| *Definite 2015* Barany Society clinical *Criteria for MD (10)* | |
|  | A. ≥2 spontaneous episodes of vertigo, each lasting 20 minutes to 12 hours  B. Audiometrically documented low- to medium-frequency SNHL in affected ear on ≥1 occasion before, during or after 1 of the episodes of vertigo  C. Fluctuating aural symptoms (hearing, tinnitus or fullness) in affected ear  D. Not better accounted for by another vestibular diagnosis |
|  |  |
| *Other clinical criteria for MD or hydropic ear disease (“Atypical” MD)* | |
| 2015 Ménière’s disease Criteria (10) |  |
| Probable | A. ≥2 episodes of vertigo or dizziness, each lasting 20 minutes to 24 hours  B. Fluctuating aural symptoms (hearing, tinnitus or fullness) in affected ear  C. Not better accounted for by another vestibular diagnosis |
| 1995 Ménière’s disease Criteria (11) |  |
| Certain Ménière’s disease | Definite MD, plus histopathologic confirmation |
| Definite Ménière’s disease | A. Two or more definitive spontaneous episodes of vertigo 20 minutes or longer  B. Audiometrically documented hearing loss on at least one occasion  C. Tinnitus or aural fullness in the treated ear  D. Other causes excluded |
| Probable Ménière’s disease | A. One definitive episode of vertigo  B. Audiometrically documented hearing loss on at least one occasion  C. Tinnitus or aural fullness in the treated ear  D. Other causes excluded |
| Possible Ménière’s disease | A. Episodic vertigo of the Ménière type without documented hearing loss, or  B. Sensorineural hearing loss, fluctuating or fixed, with dysequilibrium but without definitive episodes  C. Other causes excluded |
| Monosymptomatic cochlear hydrops (cMD) |  |
| 1972 Criteria (12) | Characterized solely by a fluctuating and progressive  sensorineural deafness with all auditory test results typical of  MD. Many patients notice a fullness in the ear  coincident with the sudden drop in hearing. Some subsequently  develop definitive dizzy spells, and the qualifying ‘‘cochlear’’ is discarded |
| Kimura et al (13) | Fluctuating hearing loss with single episode of vertigo, unsteadiness, or no vestibular symptoms |
| Japanese Clinical Practice Guideline of Ménière’s Disease (14) | Recurrent cochlear symptoms (eg hearing loss, tinnitus, aural fullness) without vertigo attacks with audiometrically demonstrated SNHL (usually low frequency or pan-frequency) |
| Gurkov (15) | Acute onset low tone acute low tone sudden onset SNHL |
| *Summary criteria for cochlear hydrops applied* | Fluctuating hearing loss or acute low tone sudden onset SNHL with or without aural fullness/tinnitus but no MD type vertigo (eg only single attack of vertigo alone, or with concurrent unsteadiness). |
| Monosymptomatic vestibular hydrops (vMD) |  |
| 1972 Criteria (12) | Characterized solely by definitive spells of vertigo. This is more  difficult to diagnose as there are no objective findings between spells. The diagnosis may be accepted upon exclusion of other  diseases. Some patients subsequently develop deafness, and the  qualifying ‘‘vestibular’’ is dropped |
| Kimura et al (13) | Recurrent episodic vertigo with or without fixed hearing loss |
| Japanese Clinical Practice Guideline of Ménière’s Disease (14) | Recurrent attacks of vertigo typical for MD and signs of peripheral dysfunction without accompanying fluctuating cochlear symptoms |
| *Summary criteria for vestibular hydrops applied* | MD type vertigo/episodic vertigo with or without aural fullness/tinnitus and with or without fixed SNHL (but not fluctuating or low frequency). |
| *Control ears* | |
|  |  |
|  | Neither ear satisfying clinical criteria for MD on any previous classification or any features of hydropic ear disease (as above) |
|  | Audiogram: Normal hearing (thresholds ≤20dBHL at 0.5, 1, 2 and 4 kHz) or isolated high frequency sensorineural hearing loss (≥20dBHL at ≥ 6 kHz) |
|  | No MD type vertigo (recurrent episodic and lasting 20 minutes to 24 hours) |
|  | Aural fullness or tinnitus permitted if it could be explained by an alternative diagnosis (e.g eustachian tube dysfunction) |

MD = Ménière’s disease

SNHL = sensorineural hearing loss

**Supplementary Table 1: Clinical criteria used to define Ménière’s disease**

|  | **Nakashima (19)** | **Barath (17)** | **Bernaerts (7)** | **Kahn (18)** |
| --- | --- | --- | --- | --- |
| **Cochlea** |  |  |  |  |
| Plane and location | **Axial**: Mid-modiolar level | **Axial:** Mid-modiolar level | **Axial:** Mid-modiolar level | **Axial:** Mid-modiolar level |
| Imaging feature/grading | *Grade 1*: cochlear duct area < scala vestibuli area  *Grade 2*: cochlear duct > scala vestibuli area* | *Grade 1*: cochlear duct spares part of scala vestibuli  *Grade 2:* cochlear duct replaces scala vestibuli* | *Grade 1:* Nodular cochlear duct enlargement “Xmas tree balls”  *Grade 2:*  Linear cochlear duct enlargement “Xmas garlands” | Cochlear duct > scala vestibuli area |
| **Vestibule** |  |  |  |  |
| Plane and location | **Axial:**  Inferior aspect of lateral semi-circular canal | **Axial:**  At widest part of the vestibule (inferior) | **Axial**:  At widest part of the vestibule (inferior) | **Axial**:  Variable  (superior and inferior vestibule) |
| Imaging feature/grading | *Grade 1:* % area of endolymph relative to total fluid area  33-50 %  *Grade 2:* % area of endolymph relative to total fluid area  >50% | *Grade 1:* >50% area of endolymph relative to total fluid area  *Grade 2:* enhancing perilymphatic space of the vestibule not visible | *Grade 1:* SURI  *Grade 2:* utricle and saccule are confluent  Grade 3: enhancing perilymphatic space of the vestibule not visible | **Saccule:**  *Grade 1:* SURI  Grade 2: touching oval window  **Utricle:**  *Grade 1:* herniation to lateral SCC  *Grade 2:* enhancing perilymphatic space of the vestibule not visible  **Ampulla:** No surrounding perilymphatic space |

**Supplementary Table 2: Description of MRI grading scales incorporating the MRI descriptors**

| **Descriptor** | **Corresponding grading scale** |  | **Details of analysis** |
| --- | --- | --- | --- |
| **Cochlear** | | | |
| Grade 1 | Barath (17)/  Bernaerts (7) | Nodular cochlear duct enlargement with cochlear duct sparing part of scala vestibuli | Axial through mid-modiolar level |
| Grade 2 |  | Linear cochlear duct enlargement with cochlear duct replacing scala vestibuli |  |
| Asymmetric PLE | NA | Asymmetrically increased PLE | Through inferior segment of basal turns |
| **Superior vestibule** | | | |
| >33% VES area relative to TV area (superior) | Nakashima (19) | There is 33-50 % area of the combined VES relative to the TV fluid area on an axial cross section | Axial parallel to and at the inferior aspect of lateral semi-circular canal where it is visualised more than 240^0^. |
| >50% VES area relative to TV area (superior) |  | There is >50 % area of the combined VES relative to TV fluid area |  |
| No lateral SCC ampullary PS visible | Kahn (18) | The extension of the VES into the ampulla of the lateral SCC is dilated so no PS is visible | Axial parallel to and at the level of the lateral semi-circular canal |
| Lateral SCC posterior limb VES extension |  | There is protrusion of the VES into the non-ampullated posterior limb of the lateral SCC |  |
| PS not visible (superior) |  | The VES replaces the TVS and no PS is visible |  |
| **Inferior vestibule** | | | |
| Saccule as large as the utricle | Bernaerts (7)/  Kahn (18)  as described by  Attyé et al (20) | Termed “SURI”. The ratio of the area of the saccule to the area of the utricle is ≥ 1 | Axial through the widest part of the vestibule and oblique sagittal in line of the vestibule |
| Saccule confluent with the utricle | Bernaerts(7) | There is confluence of the saccule and utricle (with no acute angle at its interface) | Axial angled from posterior limb of lateral semi-circular to modiolus through the widest part of the inferior vestibule |
| Absent saccule | NA | No visible saccule at its normal location |  |
| >50% VES area relative to TV area (inferior) | Barath (17) |  |  |
| PS not visible (inferior) | Bernaerts (7) | The VES replaces the TV fluid area and no PS is visible |  |
| VES contacting the oval window | Kahn (18)  as described by  Conte et al (21) | Termed “VESCO”. The VES contacts the oval window and effaces the adjacent perilymphatic space | Axial parallel to lateral SCC and oblique sagittal planes (parallel to superior SCC) |

PLE: Perilymphatic enhancement

VES: Vestibular endolymphatic space

TV(S): Total vestibular (space)

VESCO: Vestibular endolymphatic space contacts the oval window

SURI: Saccule to utricle area ratio inversion

PS: Perilymphatic space

SCC: semi-circular canal

**Supplementary table 3: Details of analysing MRI descriptors and associated grading systems**

| **Clinical classification of atypical MD ears** | N=31 |
| --- | --- |
| cMD | 13 |
| vMD | 2 |
| cMD/possible 1995 | 2 |
| Possible 1995/probable 2015 | 7 |
| Definite 1995/probable 2015 | 7 |
| **Aetiologies in patients with secondary hydrops** | N=8 |
| Post stapedectomy for otospongiosis | 2 |
| Autoimmune inner ear disease | 1 |
| Systemic vasculitic | 2 |
| Ipsilateral delayed endolymphatic hydrops post head trauma | 2 |
| Contralateral delayed endolymphatic hydrops | 1 |
| **Clinical diagnoses in other hydropic ear symptom patients** | N=48 |
| Vestibular migraine | 14 |
| Eustachian tube dysfunction | 11 |
| Benign paroxysmal positional vertigo | 9 |
| Postural-perceptual dizziness | 5 |
| Ear infection/chronic otitis media | 2 |
| Acute vestibular or vestibulo-cochlear failure | 2 |
| Semicircular canal dehiscence | 1 |
| Non specific fluctuating tinnitus | 1 |
| Psycho-acoustic hearing loss | 1 |
| Non specific vertigo | 1 |
| Head motion provoked vertigo | 1 |

**Supplementary table 4: Clinical criteria satisfied for atypical MD ears, aetiologies in patients with secondary hydrops**

**and clinical diagnoses in other hydropic ear symptoms**

| **Grading Scale** | **Cohens Kappa (95% CI)**  **ZPE** | **Cohens Kappa (95% CI)**  **NSE** | **Chi sq for association and**  **Likelihood ratio for definite MD ZPE** | **Chi sq for association and**  **Likelihood ratio for definite MD NSE** | **Chi sq for association and Cramer’s V for ZPE v NSE** |
| --- | --- | --- | --- | --- | --- |
| Nakashima (19) | *κ* =0.804 (0.739-0.868) | *κ* =0.846 (0.792-0.899) | χ^2^*=43.084*  *P<0.001*  *Likelihood ratio*  *55.605* | χ^2^*=48.252*  *P<0.001*  *Likelihood ratio 62.305* | χ^2^ =241.368  P<0.001  Cramer’s V=0.676 |
| Barath (17) | *κ* =0.910 (0.851-0.969) | *κ* =0.879 (0.827-0.932) | χ^2^ *=44.107*  *P<0.001*  *Likelihood ratio 57.543* | χ^2^*=57.080*  *P<0.001*  *Likelihood ratio 72.989* | χ^2^ =277.274  P<0.001  Cramer’s V=0.725 |
| Bernaerts (7) | *κ* =0.898 (0.850-0.946) | *κ* =0.928 (0.890-0.966) | χ^2^*=50.233*  *P<0.001*  *Likelihood ratio 64.828* | χ^2^*=57.313*  *P<0.001*  *Likelihood ratio 73.531* | χ^2^ =382.986  P<0.001  Cramer’s V=0.695 |
| Kahn (18) | *κ* =0.893 (0.850-0.937) | *κ* =0.885 (0.843-0.927) | χ^2^*=57.080*  *P<0.001*  *Likelihood ratio 72.989* | χ^2^*=55.912*  *P<0.001*  *Likelihood ratio 69.246* | χ^2^ =416.234  P<0.001  Cramer’s V=0.562 |
| Cochlear grading (7) | *κ* =0.797 (0.736-0.858) | *κ* =0.850 (0.800-0.901) | χ^2^*=56.202*  *P<0.001*  *Likelihood ratio 72.002* | χ^2^*=63.264*  *P<0.001*  *Likelihood ratio 80.693* | χ^2^ =250.209  P<0.001  Cramer’s V=0.688 |

**Supplementary table 5:**

**Inter-rater reliability Kappa values, Cramer V values for the MRI grading scales with ZPE and NPE sequences**

|  | Chi sq for association and Phi for ZPE v NSE |
| --- | --- |
| Grade ≥1 cochlear hydrops | χ^2^ =118.730  P<0.001  φ=0.671 |
| Grade 2 cochlear hydrops | χ^2^ =220.836  P<0.001  φ=0.915 |
| Asymmetric PLE | Standardised windowing*  χ^2^ =185.272  P<0.001  φ=0.838  Equivalent windowing*  χ^2^ =191.791  P<0.001  φ=0.852 |
| >33% VES area relative to TV area (superior) | χ^2^ =115.295  P<0.001  φ=0.661 |
| >50% VES area relative to TV area (superior) | χ^2^ =199.217  P<0.001  φ=0.869 |
| No lateral SCC ampullary PS visible | χ^2^ =91.986  P<0.001  φ=0.590 |
| Lateral SCC posterior limb ES extension | χ^2^ =153.280  P<0.001  φ=0.762 |
| PS not visible (superior) | χ^2^ =77.087  P<0.001  φ=0.540 |
| Saccule large as or confluent with the utricle | χ^2^ =179.667  P<0.001  φ=0.825 |
| Saccule absent, large as or confluent with the utricle | χ^2^ =184.152  P<0.001  φ=0.835 |
| Saccule confluent with the utricle | χ^2^ =196.692  P<0.001  φ=0.863 |
| >50% VES area relative to TV area (inferior) | χ^2^ =200.109  P<0.001  φ=0.872 |
| PS not visible (inferior) | χ^2^ =138.168  P<0.001  φ=0.723 |
| VES contacting the oval window | χ^2^ =206.433  P<0.001  φ=0.884 |

PLE: Perilymphatic enhancement

VES: Vestibular endolymphatic space

SCC: semi-circular canal

PS: Perilymphatic space

TV: Total vestibular

VESCO: Vestibular endolymphatic space contacts the oval window

**Supplementary table 6: Chi sq for association and Phi values for the 14 MRI descriptors with ZPE and NPE sequences**


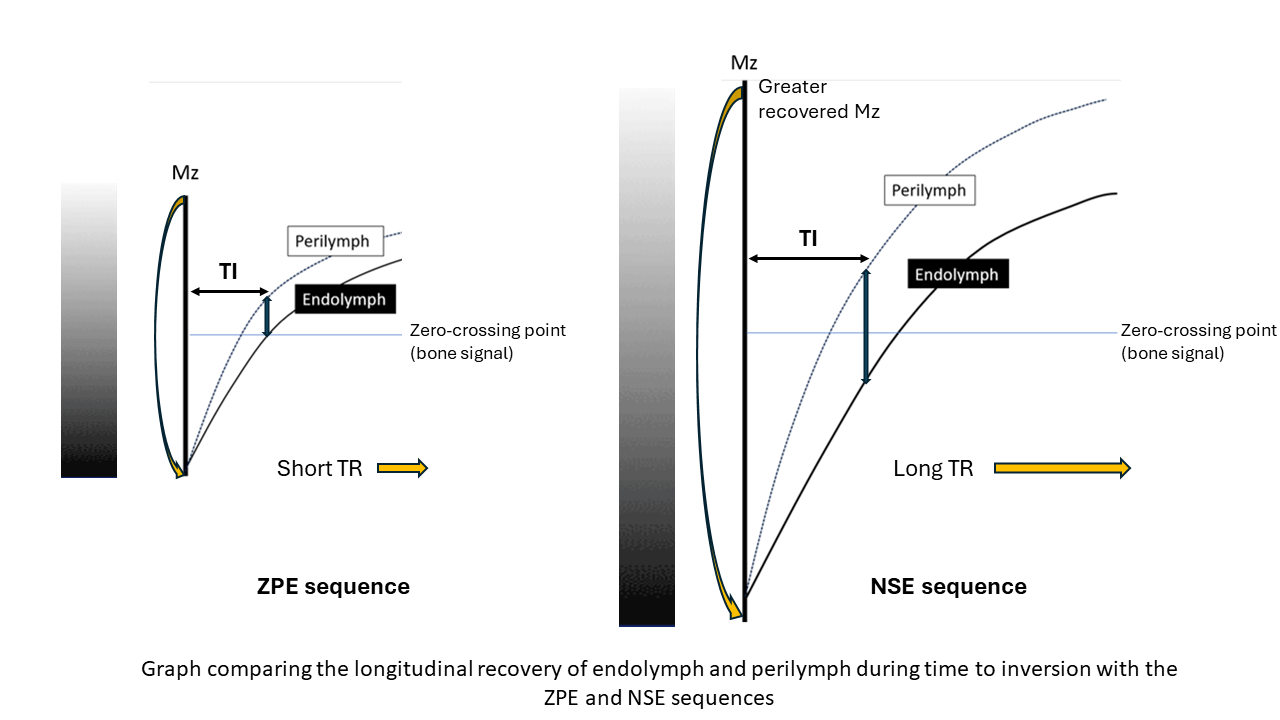


**Supplementary figure 1**
